# Supplementary material for: Dietary regimens appear to possess significant effects on the development of combined antiretroviral therapy (cART)-associated metabolic syndrome
Source: PLoS One. 2024 Feb 28;19(2):e0298752. doi: 10.1371/journal.pone.0298752 (PMC10901320; doi:10.1371/journal.pone.0298752)
Supplement: S1 File — (PDF) [file pone.0298752.s001.pdf]

Mean weekly body weight during diet induction phase

Week	Standard diet	Normal protein high calorie diet	Low protein high calorie diet
0	146.765	143.5025	145.505
1	150.0925	153.0075	151.515
2	157.8308	162.9436	161.5821
3	168.3625	172.345	174.0625
4	179.1475	181.71	182.57
5	183.425	196.7925	198.56
6	188.7425	216.01	219.275
7	197.015	230.6725	233.3275
8	208.775	255.4125	258.4925
9	218.2575	277.43	283.0675
10	221.1725	286.825	292.8325
11	228.595	301.97	307.755
12	237.0175	322.2775	329.7925
13	246.5725	341.235	352.2075
14	256.3775	360.695	372.33
15	273.0725	389.9625	398.0875
