# Supplementary material for: Dietary regimens appear to possess significant effects on the development of combined antiretroviral therapy (cART)-associated metabolic syndrome
Source: PLoS One. 2024 Feb 28;19(2):e0298752. doi: 10.1371/journal.pone.0298752 (PMC10901320; doi:10.1371/journal.pone.0298752)
Supplement: S2 File — (PDF) [file pone.0298752.s002.pdf]

Mean weekly fasting blood glucose levels during the induction phase

Week	Standard Diet	Normal Protein High Calorie Diet	Low Protein High Calorie Diet
0	3.855	3.85	3.8525
1	3.895	3.905	3.91
2	3.92	3.9625	3.99
3	3.94	3.9875	3.995
4	4.035	4.0325	4.0575
5	3.961538	4.079487	4.082051
6	4.045	4.14	4.195
7	3.985	4.1775	4.225
8	4.023077	4.228205	4.315385
9	4.0875	4.37	4.425
10	4.105	4.45	4.545
11	4.0975	4.5725	4.855
12	4.085	5.0975	5.335
13	4.145	5.6525	5.965
14	4.045	5.72	6.2575
15	4.045	5.8325	6.56
