# Supplementary material for: Dietary regimens appear to possess significant effects on the development of combined antiretroviral therapy (cART)-associated metabolic syndrome
Source: PLoS One. 2024 Feb 28;19(2):e0298752. doi: 10.1371/journal.pone.0298752 (PMC10901320; doi:10.1371/journal.pone.0298752)
Supplement: S3 File — (PDF) [file pone.0298752.s003.pdf]

Oral Glucose tolerance test at week 15

Time (Minutes)	Standard Diet	Normal Protein High Calorie Diet	Low Protein High Calorie Diet
0	4.045	5.8325	6.56
30	4.2825	6.1475	6.95
60	4.89	6.9575	8.155
90	4.4475	6.495	8.335
120	4.0975	6.1925	8.4225
