# Supplementary material for: Dietary regimens appear to possess significant effects on the development of combined antiretroviral therapy (cART)-associated metabolic syndrome
Source: PLoS One. 2024 Feb 28;19(2):e0298752. doi: 10.1371/journal.pone.0298752 (PMC10901320; doi:10.1371/journal.pone.0298752)
Supplement: S4 File — (PDF) [file pone.0298752.s004.pdf]

**Area under the curve:Week 15**

| Standard Diet | Normal Protein High Calorie Diet | Low Protein High Calorie Diet |
|---|---|---|
| 538.5 | 769.5 | 933 |
| 562.5 | 769.5 | 883.5 |
| 519 | 766.5 | 918 |
| 514.5 | 793.5 | 913.5 |
| 535.5 | 786 | 934.5 |
| 499.5 | 780 | 949.5 |
| 490.5 | 769.5 | 909 |
| 517.5 | 754.5 | 910.5 |
| 511.5 | 763.5 | 931.5 |
| 525 | 748.5 | 904.5 |
| 531 | 709.5 | 918 |
| 552 | 781.5 | 930 |
| 538.5 | 777 | 937.5 |
| 514.5 | 795 | 909 |
| 526.5 | 810 | 936 |
| 544.5 | 802.5 | 933 |
| 564 | 795 | 897 |
| 523.5 | 801 | 919.5 |
| 535.5 | 778.5 | 940.5 |
| 535.5 | 751.5 | 921 |
| 531 | 696 | 907.5 |
| 541.5 | 696 | 942 |
| 541.5 | 750 | 928.5 |
| 514.5 | 748.5 | 948 |
| 523.5 | 771 | 934.5 |
| 555 | 778.5 | 909 |
| 532.5 | 780 | 934.5 |
| 529.5 | 753 | 946.5 |
| 526.5 | 754.5 | 939 |
| 526.5 | 769.5 | 954 |
| 528 | 757.5 | 919.5 |
| 532.5 | 778.5 | 948 |
| 529.5 | 756 | 934.5 |
| 538.5 | 756 | 951 |
| 526.5 | 792 | 969 |
| 535.5 | 799.5 | 933 |
| 526.5 | 766.5 | 900 |
| 535.5 | 775.5 | 924 |
| 532.5 | 778.5 | 939 |
| 543 | 775.5 | 927 |
