# Supplementary material for: Dietary regimens appear to possess significant effects on the development of combined antiretroviral therapy (cART)-associated metabolic syndrome
Source: PLoS One. 2024 Feb 28;19(2):e0298752. doi: 10.1371/journal.pone.0298752 (PMC10901320; doi:10.1371/journal.pone.0298752)
Supplement: S5 File — (PDF) [file pone.0298752.s005.pdf]

Mean weekly body weights (standard diet) during the treatment phase

Week	Normal saline	Test group 1	Test group 2	Positive Control
16	277.17	284.37	286.61	287.97
17	292.37	293.77	301.23	302.01
18	313.05	315.42	318.51	320.4
19	319.85	318.02	320.52	322.05
20	329.37	327.13	330.49	330.77
21	339.32	340.95	342.15	343.62
22	345.73	346.79	351.14	349.64
23	359.95	360.57	364.3	362.92
24	374.82	375.24	378.56	377.13
