# Supplementary material for: Dietary regimens appear to possess significant effects on the development of combined antiretroviral therapy (cART)-associated metabolic syndrome
Source: PLoS One. 2024 Feb 28;19(2):e0298752. doi: 10.1371/journal.pone.0298752 (PMC10901320; doi:10.1371/journal.pone.0298752)
Supplement: S9 File — (PDF) [file pone.0298752.s009.pdf]

Mean weekly fasting blood glucose levels for NPHC diet group during treatment phase

Week	Normal saline	Test group 1	Test group 2	Positive control
16	5.76	5.78	5.84	6.01
17	5.89	5.91	6.04	6.08
18	6.05	6.06	6.26	6.3
19	6.47	6.5	6.67	6.69
20	6.42	6.43	6.81	6.82
21	6.61	6.58	7	7.15
22	6.77	6.72	7.11	7.2
23	6.88	6.9	7.31	7.36
24	6.94	6.97	7.41	7.45
