# Supplementary material for: Dietary regimens appear to possess significant effects on the development of combined antiretroviral therapy (cART)-associated metabolic syndrome
Source: PLoS One. 2024 Feb 28;19(2):e0298752. doi: 10.1371/journal.pone.0298752 (PMC10901320; doi:10.1371/journal.pone.0298752)
Supplement: S11 File — (PDF) [file pone.0298752.s011.pdf]

Oral glucose tolerance test for the standard diet group during the treatment phase

Time (Minutes)	Normal Saline	Test group 1	Test group 2	Positive Control
0	4.16	4.13	4.17	4.21
30	4.76	4.74	4.73	4.79
60	6.76	6.86	6.84	6.9
90	5.54	5.74	5.62	5.67
120	4.27	4.45	4.28	4.32
