# Supplementary material for: Dietary regimens appear to possess significant effects on the development of combined antiretroviral therapy (cART)-associated metabolic syndrome
Source: PLoS One. 2024 Feb 28;19(2):e0298752. doi: 10.1371/journal.pone.0298752 (PMC10901320; doi:10.1371/journal.pone.0298752)
Supplement: S12 File — (PDF) [file pone.0298752.s012.pdf]

Oral glucose tolerance test for the NPHC diet group during the treatment phase

Time (Minutes)	Normal Saline	Test group 1	Test group 2
0	6.94	6.97	7.41
30	7.37	7.46	8.04
60	8.01	8.09	9.03
90	7.86	7.92	8.97
120	7.76	7.9	8.82
