# Supplementary material for: Dietary regimens appear to possess significant effects on the development of combined antiretroviral therapy (cART)-associated metabolic syndrome
Source: PLoS One. 2024 Feb 28;19(2):e0298752. doi: 10.1371/journal.pone.0298752 (PMC10901320; doi:10.1371/journal.pone.0298752)
Supplement: S13 File — (PDF) [file pone.0298752.s013.pdf]

Oral glucose tolerance test for the LPHC group during the treatment phase

Time (Minutes)	Normal saline	Test group 1	Test group 2
0	8.72	8.91	9.29
30	9.1	9.16	10.06
60	9.85	9.79	11.07
90	9.47	9.53	10.99
120	9.1	9.21	10.71
