# Supplementary material for: Dietary regimens appear to possess significant effects on the development of combined antiretroviral therapy (cART)-associated metabolic syndrome
Source: PLoS One. 2024 Feb 28;19(2):e0298752. doi: 10.1371/journal.pone.0298752 (PMC10901320; doi:10.1371/journal.pone.0298752)
Supplement: S14 File — (PDF) [file pone.0298752.s014.pdf]

Area under the curve for the standard diet group during the treatment phase

Normal Saline	Test group 1	Test group 2	Positive Control
634.5	636	639	649.5
631.5	648	642	663
646.5	649.5	652.5	664.5
645	649.5	652.5	634.5
622.5	654	636	640.5
639	657	633	663
627	643.5	654	646.5
649.5	640.5	637.5	648
646.5	646.5	663	651
640.5	639	615	627
