# Supplementary material for: Dietary regimens appear to possess significant effects on the development of combined antiretroviral therapy (cART)-associated metabolic syndrome
Source: PLoS One. 2024 Feb 28;19(2):e0298752. doi: 10.1371/journal.pone.0298752 (PMC10901320; doi:10.1371/journal.pone.0298752)
Supplement: S15 File — (PDF) [file pone.0298752.s015.pdf]

Area under the curve for the LPHC group during the treatment phase

Normal Saline	Test group 1	Test group 2	Positive Control
1110	1128	1242	1287
1111.5	1134	1317	1299
1120.5	1126.5	1245	1291.5
1089	1096.5	1242	1245
1126.5	1114.5	1275	1301
1137	1144.5	1308	1297.5
1125	1134	1255.5	1298
1153.5	1144.5	1249.5	1270.5
1108.5	1107	1266	1270.5
