# Supplementary material for: Dietary regimens appear to possess significant effects on the development of combined antiretroviral therapy (cART)-associated metabolic syndrome
Source: PLoS One. 2024 Feb 28;19(2):e0298752. doi: 10.1371/journal.pone.0298752 (PMC10901320; doi:10.1371/journal.pone.0298752)
Supplement: S16 File — (PDF) [file pone.0298752.s016.pdf]

Area under the curve for the NPHC group during the treatment phase

Normal saline	Test group 1	Test group 2	Positive control
916.5	922.5	1014	1021.5
910.5	937.5	1009.5	1038
909	921	1039.5	1014
919.5	939	1012.5	1024.5
921	918	1044	1018.5
922.5	921	1032	1003.5
904.5	915	997.5	1018.5
919.5	933	1038	1038
915	940.5	1035	1038
