# Supplementary material for: Dietary regimens appear to possess significant effects on the development of combined antiretroviral therapy (cART)-associated metabolic syndrome
Source: PLoS One. 2024 Feb 28;19(2):e0298752. doi: 10.1371/journal.pone.0298752 (PMC10901320; doi:10.1371/journal.pone.0298752)
Supplement: S17 File — (PDF) [file pone.0298752.s017.pdf]

**Serum triglyceride for standard diet group during the treatment phase**

| Normal saline | Test group 1 | Test group 2 | Positive control |
|---|---|---|---|
| 0.91 | 0.9 | 0.87 | 1.28 |
| 1.01 | 0.83 | 1.12 | 0.86 |
| 0.76 | 0.89 | 1.1 | 0.89 |
| 0.87 | 0.67 | 0.97 | 0.93 |
| 0.93 | 1.1 | 0.97 | 1.13 |
| 0.92 | 0.56 | 0.86 | 0.94 |
| 1.21 | 0.86 | 1.14 | 1.27 |
| 0.87 | 0.74 | 0.78 | 0.74 |
| 0.87 | 0.82 | 1.25 | 0.89 |
| 1.24 | 0.73 | 0.89 | 0.88 |
