# Supplementary material for: Dietary regimens appear to possess significant effects on the development of combined antiretroviral therapy (cART)-associated metabolic syndrome
Source: PLoS One. 2024 Feb 28;19(2):e0298752. doi: 10.1371/journal.pone.0298752 (PMC10901320; doi:10.1371/journal.pone.0298752)
Supplement: S19 File — (PDF) [file pone.0298752.s019.pdf]

HDL for standard diet group during the treatment phase

Normal saline	Test group 1	Test group 2	Positive control
0.81	0.92	0.48	0.56
0.79	0.68	0.62	0.51
0.56	0.74	0.65	0.61
0.7	0.76	0.72	0.76
0.73	0.73	0.61	0.66
0.81	0.61	0.71	0.62
0.66	0.65	0.69	0.61
0.95	0.71	0.75	0.74
0.72	0.72	0.53	0.64
0.52	0.78	0.62	0.72
