# Supplementary material for: Dietary regimens appear to possess significant effects on the development of combined antiretroviral therapy (cART)-associated metabolic syndrome
Source: PLoS One. 2024 Feb 28;19(2):e0298752. doi: 10.1371/journal.pone.0298752 (PMC10901320; doi:10.1371/journal.pone.0298752)
Supplement: S20 File — (PDF) [file pone.0298752.s020.pdf]

Total cholesterol for NPHC group during the treatment phase

Normal saline	Test group 1	Test group 2	Positive control
2.41	2.36	6.12	6.11
2.86	2.65	5.89	5.86
3.16	2.87	6.13	6.13
2.76	2.67	6.21	6.02
3.32	2.33	5.98	5.67
2.67	3	6.34	6.21
2.76	2.65	5.94	6.12
3.11	2.76	5.96	5.97
2.89	2.71	6.13	6.06
2.78	2.63	5.89	5.98
