# Supplementary material for: Dietary regimens appear to possess significant effects on the development of combined antiretroviral therapy (cART)-associated metabolic syndrome
Source: PLoS One. 2024 Feb 28;19(2):e0298752. doi: 10.1371/journal.pone.0298752 (PMC10901320; doi:10.1371/journal.pone.0298752)
Supplement: S22 File — (PDF) [file pone.0298752.s022.pdf]

Total serum cholesterol for standard diet group during the treatment phase

Normal saline	Test group 1	Test group 2	Positive control
1.31	1.61	1.58	1.83
1.92	1.54	1.73	1.87
1.32	1.63	2	1.45
2	1.6	1.86	1.76
1.54	1.53	1.8	1.86
1.73	1.68	1.86	1.85
1.73	1.64	1.73	1.87
1.67	1.61	1.65	1.84
1.56	1.62	1.76	1.72
1.86	1.56	1.79	1.61
