# Supplementary material for: Dietary regimens appear to possess significant effects on the development of combined antiretroviral therapy (cART)-associated metabolic syndrome
Source: PLoS One. 2024 Feb 28;19(2):e0298752. doi: 10.1371/journal.pone.0298752 (PMC10901320; doi:10.1371/journal.pone.0298752)
Supplement: S28 File — (PDF) [file pone.0298752.s028.pdf]

Retroperioneal adipose tissue for the standard diet group

Normal saline	Test group 1	Test group 2	Positive control
8.2	7.9	8.9	8.7
7.9	8.1	8.7	8.2
8	8.2	8.3	8
8.2	8	8.2	8.1
7.7	7.9	8	8.3
8.2	8.1	8.7	8.2
8.4	8	7.9	8.6
8.2	8.3	8.1	8.1
8.1	8.1	8.4	7.8
8.2	8.2	8.6	8.8
