# Supplementary material for: Dietary regimens appear to possess significant effects on the development of combined antiretroviral therapy (cART)-associated metabolic syndrome
Source: PLoS One. 2024 Feb 28;19(2):e0298752. doi: 10.1371/journal.pone.0298752 (PMC10901320; doi:10.1371/journal.pone.0298752)
Supplement: S30 File — (PDF) [file pone.0298752.s030.pdf]

Pericardial adipose tissue for the standard diet group

Normal saline	Test group 1	Test group 2	Positive control
3	2.9	3.1	3.1
2.7	2.6	2.8	2.8
2.9	2.8	2.9	2.9
2.7	2.7	3	2.9
3	3	2.9	3.1
2.8	2.8	2.8	3
2.8	2.9	2.9	2.7
2.6	2.6	3.1	2.8
2.8	2.7	2.8	3.1
2.9	2.6	2.8	2.8
