# Supplementary material for: Dietary regimens appear to possess significant effects on the development of combined antiretroviral therapy (cART)-associated metabolic syndrome
Source: PLoS One. 2024 Feb 28;19(2):e0298752. doi: 10.1371/journal.pone.0298752 (PMC10901320; doi:10.1371/journal.pone.0298752)
Supplement: S33 File — (PDF) [file pone.0298752.s033.pdf]

Pericardial adipose tissue for NPHC diet during the treatment phase

Normal saline	Test group 1	Test group 2	Positive control
4.1	4.1	7.8	8.6
4.5	4.5	8.1	8.1
4.3	4.1	8.3	8.3
4.4	4.5	8.4	7.9
4.2	4.4	8.7	8.2
4.6	4	8.9	8.3
4.8	4.1	8.2	8
4.4	4.6	8.5	8.6
4.8	4.3	8.3	8.1
4.2	4.1	8.3	8.3
