# Supplementary material for: Dietary regimens appear to possess significant effects on the development of combined antiretroviral therapy (cART)-associated metabolic syndrome
Source: PLoS One. 2024 Feb 28;19(2):e0298752. doi: 10.1371/journal.pone.0298752 (PMC10901320; doi:10.1371/journal.pone.0298752)
Supplement: S34 File — (PDF) [file pone.0298752.s034.pdf]

**Retroperitoneal adipose tissue for LPHC diet during the treatment phase**

| Normal saline | Test group 1 | Test group 2 | Positive control |
|---|---|---|---|
| 14.7 | 14.3 | 17.8 | 17.9 |
| 14.5 | 14.5 | 17.9 | 17.4 |
| 14.8 | 14.9 | 17.3 | 17.6 |
| 14.6 | 15.1 | 17.4 | 17.1 |
| 14.2 | 14.5 | 17.6 | 17.9 |
| 14.3 | 14.8 | 17.3 | 17.4 |
| 14.1 | 14.9 | 17.2 | 17.3 |
| 14.9 | 14.1 | 17.9 | 17.2 |
| 13.8 | 13.8 | 17.5 | 17.1 |
| 14.7 | 14.3 | 17.2 | 17.9 |
