# Supplementary material for: Dietary regimens appear to possess significant effects on the development of combined antiretroviral therapy (cART)-associated metabolic syndrome
Source: PLoS One. 2024 Feb 28;19(2):e0298752. doi: 10.1371/journal.pone.0298752 (PMC10901320; doi:10.1371/journal.pone.0298752)
Supplement: S36 File — (PDF) [file pone.0298752.s036.pdf]

Pericardial adipose tissue for LPHC diet during the treatment phase

Normal saline	Test group 1	Test group 2	Positive control
6.1	6	9.2	9.4
6.7	6.8	9	9.6
6.9	6.3	9.4	9.1
6.6	6.1	9.1	9.5
6.1	6.1	9.6	9.9
6.4	6.2	9.4	9.1
6.3	5.8	9.7	10.3
6.4	6.2	9.9	9.1
6.6	6.3	9.1	9
6.2	6.3	9.7	9.6
