# Supplementary material for: Dietary regimens appear to possess significant effects on the development of combined antiretroviral therapy (cART)-associated metabolic syndrome
Source: PLoS One. 2024 Feb 28;19(2):e0298752. doi: 10.1371/journal.pone.0298752 (PMC10901320; doi:10.1371/journal.pone.0298752)
Supplement: S45 File — (PDF) [file pone.0298752.s045.pdf]

Fasting insulin levels for LPHC diet group during the treatment phase

Normal saline	Test group 1	Test group 2	Positive control
7.3	8.1	16.9	16.1
7.5	9.2	15.7	18.4
8.1	8.7	16.7	19.3
8.7	9.3	17.4	18.5
9	8.7	18.1	18.5
7.9	7.8	17.7	18.2
8.2	9.2	16.9	17.2
9.3	9.6	17.4	17.5
9.1	8.3	18.6	18.1
9	9.5	17.6	18.6
