# Supplementary material for: Dietary regimens appear to possess significant effects on the development of combined antiretroviral therapy (cART)-associated metabolic syndrome
Source: PLoS One. 2024 Feb 28;19(2):e0298752. doi: 10.1371/journal.pone.0298752 (PMC10901320; doi:10.1371/journal.pone.0298752)
Supplement: S46 File — (PDF) [file pone.0298752.s046.pdf]

Fasting insulin levels for standard diet group during the treatment phase

Normal saline	Test group 1	Test group 2	Positive control
4.2	4.5	4.4	4.3
4.3	5.1	4.8	4.3
5.1	4.7	5.1	5.1
3.2	4.6	5.2	4.3
4.8	5.1	4.6	5.4
3.1	5.4	4.7	5.1
4.7	4.8	5.1	4.8
5	5.4	5.8	5.5
5.6	3.8	4.5	4.9
4.2	4.1	5.5	5.4
