# Supplementary material for: Dietary regimens appear to possess significant effects on the development of combined antiretroviral therapy (cART)-associated metabolic syndrome
Source: PLoS One. 2024 Feb 28;19(2):e0298752. doi: 10.1371/journal.pone.0298752 (PMC10901320; doi:10.1371/journal.pone.0298752)
Supplement: S47 File — (PDF) [file pone.0298752.s047.pdf]

**HOMA B for LPHC diet group during the treatment phase**

| Normal saline | Test group 1 | Test group 2 | Positive control |
|---|---|---|---|
| 13.88 | 15.34 | 32.46 | 30.39 |
| 14.15 | 17.41 | 32.18 | 35.65 |
| 14.91 | 16.5 | 33.2 | 37.13 |
| 16.97 | 17.88 | 35.6 | 37.16 |
| 16.72 | 16.5 | 35.01 | 34.64 |
| 14.66 | 14.23 | 33.38 | 34.42 |
| 15.14 | 17.41 | 32.46 | 32.33 |
| 16.94 | 18.07 | 32.75 | 33.34 |
| 17.42 | 14.94 | 36.07 | 35.85 |
| 16.95 | 18.34 | 34.35 | 37.38 |
