# Supplementary material for: Dietary regimens appear to possess significant effects on the development of combined antiretroviral therapy (cART)-associated metabolic syndrome
Source: PLoS One. 2024 Feb 28;19(2):e0298752. doi: 10.1371/journal.pone.0298752 (PMC10901320; doi:10.1371/journal.pone.0298752)
Supplement: Attachment — Submitted filename: Response to Reviewers.docx [file pone.0298752.s056.docx]

**Editor and Reviewer comments:**

**1.****Editor:** Please ensure that your manuscript meets PLOS ONE's style requirements, including those for file naming. The PLOS ONE style templates can be found at

<https://journals.plos.org/plosone/s/file?id=wjVg/PLOSOne_formatting_sample_main_body.pdf> and

<https://journals.plos.org/plosone/s/file?id=ba62/PLOSOne_formatting_sample_title_authors_affiliations.pdf>

**Authors response:** The authors appreciate the reviewer’s comment and manuscript has been revised to meet PLOS ONE requirement,

**2.** **Editor:** We noticed you have some minor occurrence of overlapping text with the following previous publication(s), which needs to be addressed:

- <https://doi.org/10.1007/s11904-020-00483-5>

- <https://doi.org/10.1371/journal.pone.0179538>

**Authors response:** The authors agree with editor on overlapping text of the two previous publications. The authors have rephrased the paragraphs affected to correct the overlap.

**3. Editor:** In your Data Availability statement, you have not specified where the minimal data set underlying the results described in your manuscript can be found. PLOS defines a study's minimal data set as the underlying data used to reach the conclusions drawn in the manuscript and any additional data required to replicate the reported study findings in their entirety. All PLOS journals require that the minimal data set be made fully available.

**Authors response:** The authors appreciate the editor’s comment and the authors have uploaded the minimal data as Supporting information files.

**4.** **Editor:** Please review your reference list to ensure that it is complete and correct. If you have cited papers that have been retracted, please include the rationale for doing so in the manuscript text, or remove these references and replace them with relevant current references. Any changes to the reference list should be mentioned in the rebuttal letter that accompanies your revised manuscript. If you need to cite a retracted article, indicate the article’s retracted status in the References list and also include a citation and full reference for the retraction notice.

**Authors response:** The authors appreciate the editor’s comment. We have reviewed the reference list to ensure it is correct and complete. No changes have been made on the reference list.

**Reviewer #2:** A well written manuscript, but the abstract had no results. The section of the abstract titled "Methods and Results" only had methods. There was then a conclusion without having provided study results. I found no other significant issues requiring corrections. **Authors response:** The authors acknowledge the omission. We have included the study results in the abstract section.

**Reviewer #3:**
General comments
Integrase inhibitor-containing regimens should be abbreviated for easier read. A lot of your abbreviations were not given its full meaning at first mention.. (PLWH, NNRTI, INSTI, DTG, NAFLD etc). Ensure this is written in full for the sake of better understanding of concepts.
The authors would also need to be consistent with your abbreviations, "PLWH' should not be interchanged with "PWH"

**Authors response:**

(i)The authors make note of the reviewer comment on abbreviation of integrase inhibitor-containing regimen and wish to state that the integrase inhibitor-containing regimen has been abbreviated as (IICR)

(ii) The authors appreciate the reviewer’s comments and acknowledge that some abbreviations were not written in full at first mention and state that this has been done to improve clarity especially for the non-specialist readers.

**Reviewer's comment**
1. I suggest the experimental design for administering different diets (especially for phase 2) to the experimental animals be represented in tabular form for easy reference (Line 101-114)

**Authors response:** The authors note the reviewer’s suggestion and have included a paradigm illustration for easy reference.

2.The reporting of significant changes in parameters weekly, after which a table to show the same information makes it quite bulky to read. I believe a summary stating the point of significance and the number of weeks for which it was significant should suffice. While the Table can show the full information.

**Authors response:** The authors have taken note of the reviewer’s comment and have attempted to implement the suggestion. However, reporting of significant changes in parameter on weekly basis in the opinion of the authors improve clarity. Hence, the authors politely request to let the reporting of results to stay as it is.

3.“On”? Was the FBG recorded on the Call® 123 EZ II or it was an instrument/equipment used to record? If the latter is the case, then “via” should be used (Line 122)

**Authors reply**: The authors take note of the reviewer’s comments and wish to clarify that (On Call^®^ EZ II) is the trade name of the instrument (glucometer) that was used for determining the fasting blood glucose.

4.The AUC- What curve is being referred to? Curve of which graph? Where was it mentioned that a graph was plotted or was it stated in the cited reference. (line 131-132).

**Authors reply**: The authors acknowledge the reviewer’s comment. The graphs been referred to are figure 3 and figure 6.

5.The calculation (Line 139-140) looks like a screenshot cropped and pasted in the document. I suggest the calculation be re-input manually.

The authors appreciate and accept the reviewer’s comment and the formulas have been redone manually

6.Generally, the use of abbreviations needs to be worked on. The essence of using abbreviations is to help simplify continuous use of some compound terms, for clarity sake. It is noticed that some abbreviations are not defined at first mention (e.g PWLH), some are not used consistently (PWLH/PWH), and some have been previously mentioned but still get defined and mentioned concurrently (e.g AUC. see line 351 for a term that has been defined previously see line 131).

**Author response:** The authors appreciate the reviewer’s comments and acknowledge that some abbreviations were not written in full at first mention and some previously mentioned still get defined concurrently, this correction has been done to improve clarity.

Discussion: line 1010, I believe the population of sub-saharan Africa is more than 6% of the world's population, ensure your findings are made correctly. 7.Please fact check and add reference.

**Author response:** The authors admit to the oversight and have added the correct population percentage of sub-saharan which is 18% of the world’s population.
